# Genotypic and phenotypic characterization of *Mycobacterium tuberculosis* resistance against fluoroquinolones in the northeast of Iran

**DOI:** 10.1186/s12879-020-05112-5

**Published:** 2020-06-01

**Authors:** Mahdieh Sayadi, Hosna Zare, Saeed Amel Jamedar, Seyed Isaac Hashemy, Zahra Meshkat, Saman Soleimanpour, Sven Hoffner, Kiarash Ghazvini

**Affiliations:** 1 Antimicrobial Resistance Research Center, Mashhad University of Medical Sciences, Mashhad, Iran; 2 Department of Microbiology and Virology, Faculty of Medicine, Mashhad University of Medical Sciences, Mashhad, Iran; 3 Student Research Committee, Mashhad University of Medical Sciences, Mashhad, Iran; 4 Department of Clinical Biochemistry, Faculty of Medicine, Mashhad University of Medical Sciences, Mashhad, Iran; 5 Department of Global Public Health, Karolinska Institute, Stockholm, Sweden

**Keywords:** *Mycobacterium tuberculosis*, MDR-TB, Resistance, XDR-TB, Fluoroquinolone

## Abstract

**Background:**

Fluoroquinolones are broad-spectrum antibiotics that are recommended, and increasingly important, for the treatment of multidrug-resistant tuberculosis (MDR-TB). Resistance to fluoroquinolones is caused by mutations in the Quinolone Resistance Determining Region (QRDR) of *gyrA* and *gyrB* genes of *Mycobacterium tuberculosis.* In this study, we characterized the phenotypic and genotypic resistance to fluoroquinolones for the first time in northeast Iran.

**Methods:**

A total of 123 *Mycobacterium tuberculosis* isolates, including 111 clinical and 12 collected multidrug-resistant isolates were studied. Also, 19 WHO quality control strains were included in the study. The phenotypic susceptibility was determined by the proportion method on Löwenstein-Jensen medium. The molecular cause of resistance to the fluoroquinolone drugs ofloxacin and levofloxacin was investigated by sequencing of the QRDR region of the *gyrA* and *gyrB* genes.

**Results:**

Among 123 isolates, six (4.8%) were fluoroquinolone-resistant according to phenotypic methods, and genotypically three of them had a mutation at codon 94 of the *gyrA* gene (Asp→ Gly) which was earlier reported to cause resistance. All three remaining phenotypically resistant isolates had a nucleotide change in codon 95. No mutations were found in the *gyrB* gene. Five of the 19 WHO quality control strains, were phenotypically fluoroquinolone-resistant, four of them were genotypically resistant with mutations at codon 90, 91 of the *gyrA* gene and one resistant strain had no detected mutation.

**Conclusions:**

Mutation at codon 94 of the *gyrA* gene, was the main cause of fluoroquinolone resistance among *M. tuberculosis* isolates in our region. In 3/6 fluoroquinolone-resistant isolates, no mutations were found in either *gyrA* or *gyrB.* Therefore, it can be concluded that various other factors may lead to fluoroquinolone resistance, such as active efflux pumps, decreased cell wall permeability, and drug inactivation.

## Background

Tuberculosis (TB) is among the leading health problems, especially in low-income countries, and is considered as the first cause of death from an infectious disease and ninth cause of death worldwide [1–5]. The emergence of multidrug-resistant (MDR) form of this disease makes the treatment difficult even in developed countries [6]. Inappropriate use of antibiotics has led to the development of MDR-TB and extensively drug-resistant tuberculosis (XDR-TB). MDR-TB is defined as *Mycobacterium tuberculosis* strains that are resistant to the main first-line drugs (isoniazid and rifampin) [7]. XDR-TB strains are characterized by resistance to at least one of the three injectable aminoglycosides (kanamycin, amikacin, capreomycin) and fluoroquinolones (FQs), as well as isoniazid and rifampin [8]. The development of such resistant strains is a serious threat to the global control of tuberculosis [9–11].

FQs are synthetic chemotherapeutic agents that have broad antibacterial effects on mycobacteria [12]. Regarding the excellent pharmacokinetic and pharmacodynamic properties, FQs are considered as the most effective second-line drug and among the recommended therapy for MDR-TB [12–19]. Since FQs have broad-spectrum activity, they are usually overprescribed for various other bacterial infections, and they are easily accessible as over-the-counter medications in many resource-limited countries, exacerbating their misuse [18, 20]. This has led to the increased emergence of FQ-resistant *M. tuberculosis* strains [21, 22]. Resistance can also be developed due to the incomplete adherence to the TB treatment program, failure in previous treatment and contact with patients of resistant tuberculosis [18].

The FQ resistance is due to mutations of the quinolone resistance determining region (QRDR) in the *gyrA* and *gyrB* genes of *M. tuberculosis* [23], most common by mutation in codon 90, 91, and 94 of the *gyrA* gene. In a minority of cases, FQ resistance has been shown to be associated with mutations in the *gyrB* gene [24].

According to WHO reports in 2017, the global proportion of MDR-TB/rifampin-resistant TB (RR-TB) with resistance to any of the FQs ofloxacin, levofloxacin, and moxifloxacin was 20% [23, 25, 26]. Drug resistance of *M. tuberculosis* is an essential obstacle for effective treatment and control of TB. Therefore, timely detection of drug-resistant TB (DR-TB), including FQs, is necessary to reduce the transmission and to initiate effective drug therapy without any delay [27].

In this study, the resistance of *M. tuberculosis* against ofloxacin and levofloxacin was investigated by phenotypic and genotypic diagnostic methods for the first time in Northeastern Iran. This region has high importance regarding the TB prevalence due to migration from neighboring countries [28]. We selected a drug from the second-line anti-TB treatment (ofloxacin) and also a drug from the third-line (levofloxacin) and used the recommended test concentration of 2 μg/ml. The phenotypic susceptibility was determined by the proportion method on Lowenstein-Jensen medium. Genotypic susceptibility was evaluated by investigating possible resistance-related mutations by Sanger sequencing of the QRDR of *gyrA* and *gyrB* genes.

## Methods

### Sample collection

A total of 111 clinical isolates, including 109 pulmonary and two extra-pulmonary specimens, were collected from patients that referred to the Tuberculosis Regional Reference Laboratory in Mashhad. We included all confirmed TB patients referred to the hospital for 1 year (March 2017–February 2018). Confirmation was based on AFB smear tests, culture on LJ medium, chest x-ray, and clinical presentations. Also, 12 MDR-TB isolates related to the referred TB patients were obtained from the Mycobacterium Bank of the Department of Microbiology at Mashhad University of Medical Sciences during April 2014–February 2017. It should be noted that none of our 111 collected clinical isolates were MDR. Furthermore, 19 WHO quality control strains sent to the Tuberculosis Regional Reference Laboratory of Mashhad were included in this study. From the 111 collected clinical isolates (from 64 men, 47 women), we had 85 patients with primary tuberculosis (52 men, 33 women), 15 patients with relapse (five men, 10 women), eight patients under treatment (five men, three women) and three patients with treatment failure (two men, one woman). There were 103 Iranian patients and eight from neighboring countries. The 12 MDR-TB isolates (from 10 men and two women) included nine Iranian, two Afghan, and one Kazakh patient.

Specimens were digested and decontaminated by Petroff’s method, and the smear stained with Ziehl–Neelsen method. The specimens were also cultured on a Lowenstein-Jensen culture medium. Positive specimens were identified and confirmed by IS6110 PCR.

### Antimicrobial agents

Based on previous studies on the MIC of FQs in Iran and other countries, two concentrations of two antibiotics were selected. We used both concentrations of 2 and 4 μg/ml for ofloxacin and levofloxacin. As results were the same for both concentrations, the breakpoint for ofloxacin and levofloxacin (Temad, Iran) was selected as 2 μg/ml according to the WHO recommendations and our results [27]. To prepare this concentration, 20 mg of each antibiotic was dissolved in 10 ml of 0.1 N HCl, and 1 ml of the suspension was added to 9 ml of distilled water. Then, 1 ml of the above solution was filtered (0.22 μm) and added to 99 ml of autoclaved Lowenstein-Jensen culture medium after cooling (Merck, Heidelberg, Germany).

### Drug susceptibility test (DST)

Drug susceptibility test was performed by the proportion method on Lowenstein-Jensen medium. A microbial suspension containing 3*10^8^ CFU/ml was prepared (1 McFarland) and diluted to 1:100, 1:10000 and 1:100000. Then, 0.2 ml of these were added to the Lowenstein-Jensen medium and incubated at 37 °C for 28 days. The H37Rv reference strain was used as the control in the drug susceptibility test. All the strains in this study were also tested for the first-line TB drugs including isoniazid, rifampin, and ethambutol.

### DNA extraction

According to the simple boiling protocol for DNA extraction, 400 μl of deionized distilled water or 0.5% TE buffer was added into a 1.5 ml microtube, and a colony was also put in the tube. Then, it was centrifuged at 13000 g for 30 min and the supernatant was removed gently. After that, we performed two steps of washing with TE buffer (10 mM Tris-HCl, 1 mM EDTA, pH = 8) and centrifuged at 13000 g. The appropriate amount of TE buffer was added to the pellet (200–400 μl) and boiled for 30 min. Then, 13,000 g centrifugation was performed, and the supernatant containing DNA was used for molecular tests.

The sequence of *gyrA* and *gyrB* primers and the product size are shown in Table 1. The 100 μM stock solution of lyophilized primers (*Microsynth*, *Switzerland)* was prepared according to manufacturer instructions. The non-diluted primer was stored at − 70 °C and the diluted primer at − 20 °C.

**Table 1:** Nucleotide sequence of primers and the amplicon size

Target organism	Gene	Primer sequence	Amplicon size	T_m_	Reference
*M. tuberculosis*	*gyrA*	F: CAGCGCAGCTACATCGACTA	356 bp	61	[27]
		R: CTCAGCATCTCCATCGCCAA			
	*gyrB*	F: GCAACACCGAGGTCAAATC	710 bp	57	[29]
		R: ACCCTTGTACCGCTGAATG			

### Polymerase chain reaction

For *gyrA* gene amplification, a total volume of 40 μl containing 4 μl of the 10X PCR buffer, 2 μl MgCl_2_, 1 μl dNTPs, 0.4 μl PFU enzyme, 1 μl Dimethyl sulfoxide (DMSO), 5 μl of the DNA template, 2 μl of each of the primers and 22.6 μl of the deionized distilled water (DDW) was prepared. The thermal conditions were as follows: 1 cycle of 95 °C for 5 min, followed by 40 cycles of 95 °C for 35 s, 61 °C for 30 s, and 72 °C for 45 s, and 1 cycle of 72 °C for 5 min.

To amplify the *gyrB* gene, a total volume of 40 μl containing 4 μl of the 10X PCR buffer, 2 μl MgCl_2_, 1 μl dNTPs, 0.4 μl PFU enzyme, 1 μl DMSO, 5 μl of the DNA template, 2 μl of each of the primers and 22.6 μl of the DDW was prepared. The cycling conditions in thermal cycler were as follows: 1 cycle of 95 °C for 5 min, followed by 40 cycles of 95 °C for 35 s, 54 °C for 30 s, and 72 °C for 50 s, and 1 cycle of 72 °C for 10 min. To confirm the gene amplification, electrophoresis on 1.5% agarose gel was performed in the voltage of 90 V and time of 40–50 min.

### Sequencing

For sequencing by the Sanger method, 25 μl of PCR product was sent to Microsynth, Switzerland. Results of sequencing were analyzed by Chromas V 1.45, DNAMAN V 4.13, MEGA V 5.05 and Blast software at NCBI site.

## Results

### Phenotypic drug susceptibility

In this study, to measure the susceptibility against ofloxacin and levofloxacin, the proportion method was used. Six isolates (4.8%, five men and one woman) of the 123 tested samples from Iran and five isolates of the 19 WHO control strains, showed resistance to 2 μg/ml ofloxacin and levofloxacin. The results of antibiotic susceptibility tests of isolates are shown in Table 2. We had three FQ mono-resistant isolates (3/111) which mean being resistant only to FQs, but sensitive to all first-line TB drugs. These three isolates were all related to Iranian patients. Two of them were new TB cases and one was a TB relapse which was treated before with a 6-month rifampicin-based regimen 2HRZE/4HR according to the WHO treatment protocol [31].

**Table 2:** Classification of isolates of Tuberculosis Regional Reference Laboratory in Mashhad (*n* = 123) based on antibiotic susceptibility testing and distribution of gyrA and gyrB genes mutation patterns

Gene	Nucleotide change	Amino acid change	Clinical isolates (*n* = 111)		MDR-TB (*n* = 12)	Polydrug resistant (*n* = 0)
			Pansusceptible (***n*** = 108)	FQ mono-resistant (***n*** = 3)^**a**^		
*gyrA*	C(269) → T	Ala(90) → Val	–	–	–	–
	T(271) → C	Ser (91) → Pro	–	–	–	–
	A(281) → G	Asp(94) → Gly	–	1 (new case)	2 (XDR)^b^	–
*gyrB*	–	–	–	–	–	–
Phenotypically FQ resistant, without any gyrase mutations			–	2 (1 new, 1 relapse)	1	–
All phenotypically FQ resistant			–	3	3^c^	–

Note: Pansusceptible: susceptible to all first-line anti-TB drugs, FQ mono resistance: being resistant only to FQ, but sensitive to all first-line TB drugs, MDR-TB: resistance to at least both isoniazid and rifampin, Polydrug resistance: resistance to more than one first-line anti-TB drug, other than both isoniazid and rifampin

^a^These three isolates were all related to Iranian patients. Two of them were new TB cases and one was a TB relapse

^b^These two MDR isolates were resistant to FQs and also they were found to be resistant against kanamycin and amikacin in another study by our colleagues on these isolates [30]. Both were TB treatment failure cases, one from Afghanistan and one from Kazakhstan

^c^All were treatment failure cases

### Genotyping

The PCR reaction for *gyrA* and *gyrB* genes was performed on all 11 FQ-resistant isolates (3/111 clinical isolates, 3/12 MDR isolates, and 5/19 WHO control strains) and the *M. tuberculosis* H37Rv reference strain. Genotyping was done by Sanger sequencing the QRDR region of the *gyrA* and *gyrB* genes.

No mutations were found in the *gyrA* and *gyrB* genes of the drug-susceptible H37Rv strain. From the three phenotypically FQ-resistant isolates among 111 clinical isolates, one carried mutation at codon 94 (Asp→ Gly) which was an Iranian new TB case and two others had no gyrase mutation (one Iranian new TB case and one Iranian TB relapse explained before). From the three phenotypically FQ-resistant isolates among 12 MDR isolates, two of them carried mutation at codon 94 (both were TB treatment failure cases, one from Afghanistan and one from Kazakhstan) and one had no gyrase mutation (an Iranian TB treatment failure case). The G284C polymorphism encoding S95T polymorphism was detected in 100% of isolates. The frequency of nucleotide substitutions among different groups is provided in Table 2.

### Results of WHO quality control strains

From the five phenotypically FQ-resistant strains among 19 WHO strains, four were also genotypically resistant. Two cases had mutation at codon 90 (Ala→Val), and two had simultaneous mutations in codons 90 (Ala→Val) and 91 (Ser → Pro). The one remaining strain of phenotypically FQ-resistant WHO strains had no gyrase mutations. Data of WHO control strains are mentioned only to validate the phenotyping and genotyping results, and they are not related to Iranian samples. All the findings about control strains were in agreement with the WHO consensus results.

## Discussion

MDR-TB and XDR-TB are significant barriers to TB control programs, especially in low and middle-income countries. The risk of XDR-TB increases due to the inappropriate use of second-line anti-TB drugs, and the treatment of XDR-TB is challenging, time-demanding, costly, and often related to a poor treatment outcome [32]. The results of a meta-analysis for the treatment response of 6724 MDR-TB patients showed a 64% treatment success, while 48% for FQ-resistant cases. Therefore, routine screening is required to detect FQ resistance [33].

In this study, a second-line anti-TB drug (ofloxacin) and a third-line drug (levofloxacin) were evaluated for the analysis of phenotypic DST and genotypic resistance to FQs. They were examined on 111 clinical isolates of *M. tuberculosis*, 12 MDR-TB isolates, and also 19 WHO quality control strains sent to the laboratory.

The critical concentration was 2 μg/ml according to the WHO recommendation [27]. In the total 123 isolates of northeastern Iran, six isolates showed concurrent resistance against both these FQs. Three of these resistant isolates were among the 111 clinical isolates which were fully susceptible to the first-line anti-TB drugs isoniazid, rifampin, and ethambutol. This shows that FQ mono-resistance is a problem in Iran that risks the efficient treatment of MDR-TB due to the overuse of this drug for the treatment of community-acquired pneumonia and other bacterial diseases, as well as the treatment of non-TB respiratory symptoms [21, 22].

The fact that three (25%) out of 12 MDR-TB isolates were FQ-resistant, clearly shows that the incidence of XDR-TB or pre-XDR-TB in the northeast of Iran is worrisome. Timely drug susceptibility tests should be implemented to manage MDR-TB. It should be noted that two XDR samples were identified in this study among 12 MDR isolates as they were FQ-resistant and also have shown resistance against kanamycin and amikacin in another study by our colleagues on these isolates [30]. Both of the XDR-TB isolates were treatment failure cases and related to foreign nationals (Afghanistan, Kazakhstan), which indicates the effect of migration on tuberculosis epidemiology as the east border countries of Iran is a source of infection and affect the TB, and especially drug-resistant TB prevalence in Iran [28].

Rapid molecular methods facilitate the diagnosis of MDR-TB and pre-XDR-TB isolates in smear-positive samples and they should be used as the primary method to obtain early drug susceptibility results. The most commonly used molecular methods for the detection of FQ resistance include PCR, DNA sequencing, and line-probe assays.

In this study on 123 isolates, six FQ-resistant isolates were identified by the phenotypic method, while three were detected by sequencing. We observed the mutations only for *gyrA* and not for *gyrB* which is in accordance with the study by Arjomandzadegan et al. in Iran and Belarus [34], suggesting that the resistance of *M. tuberculosis* to FQs is generally caused by mutations in the *gyrA* gene [23]. Yu et al. in China reported only one (1.6%) mutated *gyrB* from 60 levofloxacin-resistant *M. tuberculosis* clinical isolates [35]. *gyrA* sequencing had a sensitivity of 50% to detect clinically relevant FQ resistance in our study. The sensitivity of the sequencing method for FQ resistance was reported as 77.5% by Chen et al. and 87.8% by Moure et al. [27, 36].

In this study, all our three genotypically resistant samples carried a mutation at codon 94, while the genotypically resistant WHO strains had mutations at codons 90 and 91. In a study by Arjomandzadegan et al. in Iran, 24/42 (57%) FQ-resistant strains of *M. tuberculosis* carried mutations at codon 94 [34]. In a report by Singh et al. in Tokyo, *gyrA* and *gyrB* mutations were reported in 79 and 5% among 100 ofloxacin-resistant isolates [37]. In a study by Singhal et al., 25/152 isolates had mutations in the *gyrA* gene with the most common mutations at the codon 94, while no mutations were reported in the *gyrB* gene [38].

We found 2.7% (3/111) FQ-resistant strains that were sensitive to all first-line drugs. Sharma et al. in India reported that the occurrence of FQ mono-resistant strains sensitive to the first-line treatment was 3.2% (35/1099) [39]. Kim et al. in Korea found 0.7% (38/5221) FQ mono-resistance [40]. The existence of FQ mono-resistant TB cases would be a warning against the widespread use of this drug.

There were 3/6 (50%) FQ-resistant isolates that did not harbor any *gyrA* or *gyrB* mutations. In a similar study by Zhang et al. in China, more than 30% of FQ-resistant strains had no mutations in the QRDRs of *gyrA* and *gyrB* [41]. It is believed that FQ-resistant strains with no gyrase mutations probably represent heteroresistance, which is the simultaneous presence of drug-susceptible and drug-resistant bacilli. The frequency of heteroresistance has been reported in 14 to 38% of FQ-resistant *M. tuberculosis* isolates [42].

This study was the first describing FQ-resistant TB in northeast Iran. We used two FQs from the two lines of TB treatment and performed both phenotypic and genotypic diagnostic methods which have not been done before in our region.

## Conclusions

In conclusion, a relationship between phenotypic resistance to FQs and mutations in QRDRs was detected among *M. tuberculosis* clinical isolates of Iran. In our study, mutation at codon 94 of the *gyrA* gene was the main cause of resistance to FQs in *M. tuberculosis* isolates. Three of the six FQ-resistant isolates showed a mutation in the *gyrA* gene, which causes resistance and the remaining three isolates had nucleotide change in the codon 95, which is not related to resistance. It suggests that different factors lead to the resistance of *M. tuberculosis* to FQs, such as mutations outside the QRDR of the *gyrA* gene or reduced cell wall permeability and efflux pump mechanisms [43]. Due to the inappropriate use of FQs, the resistance to these drugs in the northeast of Iran can be a warning and should be monitored. In our study, *gyrA* sequencing had a sensitivity of 50% for clinical resistance.

## Data Availability

All data generated or analysed during this study are included in this article.

## Abbreviations

TB: Tuberculosis
FQ: Fluoroquinolone
MDR: Multi-Drug Resistant
XDR-TB: Extensively Drug-Resistant Tuberculosis
QRDR: Quinolone Resistance Determining Region
RR-TB: Rifampin Resistant TB
DST: Drug Susceptibility Test
DMSO: Dimethyl Sulfoxide
DDW: Deionized Distilled Water
